# Continental phylogeography of an ecologically and morphologically diverse Neotropical songbird, *Zonotrichia capensis*

**DOI:** 10.1186/1471-2148-13-58

**Published:** 2013-03-01

**Authors:** Stephen C Lougheed, Leonardo Campagna, José A Dávila, Pablo L Tubaro, Darío A Lijtmaer, Paul Handford

**Affiliations:** 1 Department of Biology, Queen’s University, Kingston, ON, K7L 3N6, Canada; 2 División de Ornitología, Museo Argentino de Ciencias Naturales “Bernardino Rivadavia”, Avenida Ángel Gallardo 470, Ciudad de Buenos Aires, Buenos Aires, C1405DJR, Argentina; 3 Instituto de Investigación en Recursos Cinegéticos, Ronda de Toledo s/n, Cuidad Real, 13005, Spain; 4 Department of Biology, University of Western Ontario, London, ON, N6A 5B7, Canada

**Keywords:** Colonization, Demographic expansion, Intraspecific divergence, DNA sequences, Pleistocene, Rufous-collared sparrow

## Abstract

**Background:**

The Neotropics are exceptionally diverse, containing roughly one third of all extant bird species on Earth. This remarkable species richness is thought to be a consequence of processes associated with both Andean orogenesis throughout the Tertiary, and climatic fluctuations during the Quaternary. Phylogeographic studies allow insights into how such events might have influenced evolutionary trajectories of species and ultimately contribute to a better understanding of speciation. Studies on continentally distributed species are of particular interest because different populations of such taxa may show genetic signatures of events that impacted the continent-wide biota. Here we evaluate the genealogical history of one of the world’s most broadly-distributed and polytypic passerines, the rufous-collared sparrow (*Zonotrichia capensis*).

**Results:**

We obtained control region DNA sequences from 92 *Zonotrichia capensis* individuals sampled across the species’ range (Central and South America). Six additional molecular markers, both nuclear and mitochondrial, were sequenced for a subset of individuals with divergent control region haplotypes. Median-joining network analysis, and Bayesian and maximum parsimony phylogenetic analyses all recovered three lineages: one spanning Middle America, the Dominican Republic, and north-western South America; one encompassing the Dominican Republic, Roraima (Venezuela) and La Paz (Bolivia) south to Tierra del Fuego, Argentina; and a third, including eastern Argentina and Brazil. Phylogenetic analyses suggest that the Middle American/north-western South American clade is sister to the remaining two. Bayesian and maximum likelihood coalescent simulations used to study lineage demographic history, diversification times, migration rates and population expansion together suggested that diversification of the three lineages occurred rapidly during the Pleistocene, with negligible gene flow, leaving genetic signatures of population expansions.

**Conclusions:**

The Pleistocene history of the rufous-collared sparrow involved extensive range expansion from a probable Central American origin. Its remarkable morphological and behavioral diversity probably represents recent responses to local conditions overlying deeper patterns of lineage diversity, which are themselves produced by isolation and the history of colonization of South America.

## Background

Phylogeographic studies provide insight into the influence of geological and paleoclimatic events on contemporary species distributions and colonization history, and contribute to understanding the processes that might lead to new species [1]. Species with distributions on the scale of continents are particularly useful as they are often not at evolutionary equilibrium throughout their range, with distinct populations containing genetic signatures of events that have differentially impacted the continent-wide biota [2]. Phylogeographic research has focused on northern hemisphere temperate and boreal taxa, especially evaluating the influence of glaciation and postglacial dynamics; phylogeographic studies of taxa from the southern hemisphere are not as common [3].

While most of South America’s continental biota has not been as deeply influenced by glaciations (e.g., [4,5]; but see [6,7]), over the last several million years, the continent has experienced marked topographic, climatic and vegetational changes, even at low and mid latitudes (e.g., [8-10]), much of this associated with orogenesis at the continent’s western margins (e.g., [11-13]). The interaction of these factors has been proposed to underlie much of the hyperdiversity evident in the Neotropics (e.g., [14-17]).

**Figure 1:**
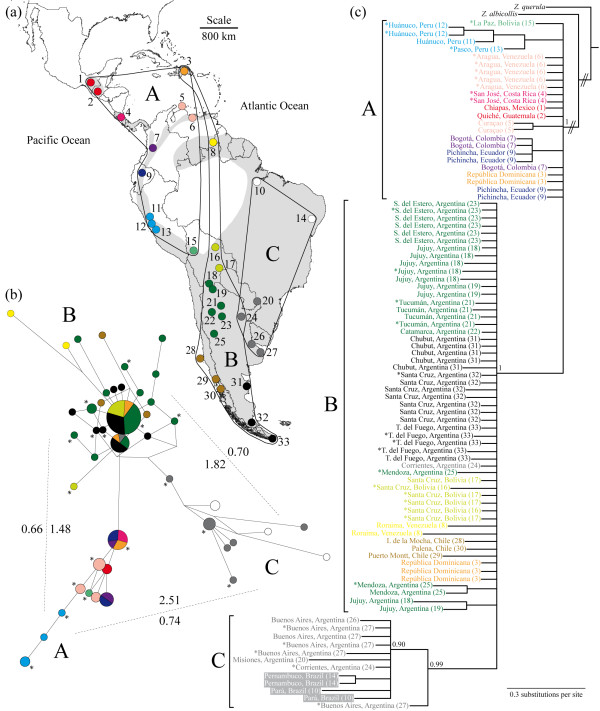
**Phylogeography of the rufous-collared sparrow inferred using mitochondrial control region sequences. (A)** Approximate geographic range of *Zonotrichia capensis* in grey (following [18]; distribution in northern South America and Central America is patchier than represented here). Sampling localities are indicated by colour-coded circles to show haplotype origin in subsequent figures. Localities are also numbered (1 to 33 from north to south); details in Table 1 follow this numerical code. Finally, the distribution of *Z. capensis* individuals belonging to the three intraspecific lineages identified is indicated with black lines (labeled **A**, **B** and **C** as in the main text). **(B)** Median-joining network showing the relationship among the 44 CR haplotypes found. Circles represent distinct haplotypes with size proportional to the number of individuals in the sample that contained it (the smallest circles represent one individual, while the largest corresponds to 23). Haplotypes are colour-coded following sampling localities. The length of the lines that connect circles is proportional to the number of mutations in which haplotypes differ. Dotted lines indicate comparisons of divergence between lineages, with values of percent p distances facing the interior of the network and those corresponding to Φ_ST_ calculations on the exterior (p < 0.001 in all cases for the latter). Asterisks show haplotypes contained by samples that were chosen for further sequencing. **(C)** Bayesian 50% majority rule consensus tree produced using 396 bp of CR sequences from the CR dataset with posterior probabilities indicating node support. *Z. capensis* individuals are represented by the locality where they were sampled (colour-coded as in Figure 1A and numerically coded as in Table 1); lineage membership is indicated. Note that Brazilian localities (coded in white) are shown with a dark background for increased contrast. Low posterior probabilities (below 0.90) were omitted for simplicity. Asterisks as in (**B**).

The rufous-collared sparrow, *Zonotrichia capensis*, is an excellent species for examining the influence of a dynamic continental history on intraspecific evolutionary patterns. It is one of the most wide-ranging New World birds, distributed from Chiapas, Mexico (10°N) to Tierra del Fuego, Argentina (55°S). It breeds in virtually all open habitats from sea level to > 4,000 metres above sea level, being absent only from continuous closed forest, including much of the Amazonian basin ( [18]; see Figure 1A). Its four congeners (*Z. albicollis*, *Z. atricapilla*, *Z. leucophrys*, and *Z. querula*) are found only in temperate North America. This distribution has been taken by Chapman [18] to imply a Nearctic origin for the genus and, accordingly, a southward expansion of the Z. *capensis* ancestor from the northern temperate zone into South America. However, the historical scenario put forward by Chapman [18] is based simply on taxon concentration and does not consider the phylogenetic relationship among *Zonotrichia* species. Phylogenetic analyses of the genus show *Z. capensis* to be sister to all North American taxa [19-21]. This suggests that the reconstruction of the distribution of the *Zonotrichia* ancestor is uncertain and thus a Central or South American origin of the genus (reflecting the range of *Z. capensis*) could also be possible. Contrary to Chapman’s scenario [18], such a Central or South American origin would imply an expansion and diversification towards the North, generating the clade found currently in North America, and the expansion of the *Z. capensis* lineage within Central and South America. Recently Barker *et al.*[22] found *Zonotrichia* to be embedded within a clade with three other exclusively North American genera: *Junco*, *Passerella* and *Spizella*; where *Junco* was sister to the remaining genera. This supports a North American origin of *Zonotrichia* and suggests that the ancestor of *Z. capensis* subsequently expanded into South America (i.e., Chapman’s 1940 scenario [18]). Regardless of the specific origin of the genus, it is likely that the ancestor of *Z. capensis* experienced many of the factors that have driven recent speciation in the Neotropics while expanding to occupy most of South America.

*Zonotrichia capensis* is one of the most polytypic avian species, with more than 20 described subspecies [18,23,24]. Morphological variation is accompanied by remarkable variation in vocal behaviour: low-latitude populations exhibit individual song repertories (e.g., Costa Rica [25]; Ecuador, P.H. personal observation), while others (~20-40°S) show individual stereotypy and geographical dialects, which correspond with natural vegetation types, but not with subspecies designations [23,26-30]. The rufous-collared sparrow also shows substantial variation in migratory habit, from lowland tropical populations that appear sedentary, through populations that undergo altitudinal migrations (e.g., altiplano populations in southern Bolivia and northern Argentina), to those that are long distance latitudinal migrants (e.g., *Z. c. australis* and *choraules* where some populations migrate more than 30° in latitude). Handford [24] showed that the majority of subspecies are only weakly differentiated morphologically. The present study will help us to understand the origins and significance of such phenotypic traits in *Z. capensis* by comparing the patterns of morphological, cultural and behavioral diversity with that of historical lineage diversification.

Previous work on genetic variation in *Z. capensis* in northwestern Argentina showed differentiation between Andean and lowland populations in allozyme frequencies [31], and in mtDNA restriction fragment length polymorphisms [32]. Moreover, Cheviron and Brumfield [33] found differences in mtDNA sequences across altitudinal transects but not across latitudinal control transects in Peruvian populations of *Z. capensis*. Cheviron *et al.*[34] reported variation in transcriptomic profiles between lowland and highland environments; however these differences were not observed when birds were transplanted to a control lowland site, suggesting plastic expression patterns that allow adaptation to high altitude conditions in this species. Significant mtDNA sequence divergence between Costa Rican and Bolivian exemplars of *Z. capensis* (2.1% in cytochrome *b*) was noted by Zink *et al.*[20] and Zink and Blackwell [21]. While these data provide provocative evidence of genetic differentiation, the aforementioned studies were too narrow in geographic scope to permit definite conclusions about the diversification history of the entire species.

Here we use mitochondrial and nuclear DNA sequences from individuals across the range of *Z. capensis* to examine the phylogeographic structure of this widely distributed emberizine songbird and address three questions:

1. What does the pattern of genetic diversification imply about the origin and historical demography of *Zonotrichia capensis*?

2. To what degree have population fragmentation and range expansion played a role in shaping genealogical patterns within the species?

3. How does phylogeographic structure relate to morphological and behavioral variation and to subspecies designations?

Our analyses revealed three main lineages within *Z. capensis* that diversified without gene flow during the Pleistocene, expanding to colonize South America from a probable Central American origin. These patterns of lineage diversity are likely the consequence of geographical isolation and the colonization of South America and do not coincide with variation reflected in subspecies designations. The remarkable morphological and behavioral diversity in the species probably represents a more recent response to local environmental conditions.

## Methods

### Specimen information, DNA extraction and sequencing

We obtained samples from 93 *Z. capensis* individuals from 33 sites across the entire breeding range (one to seven individuals per locality; Table 1, Figure 1A). We included single individuals of two congeners as outgroups: *Zonotrichia querula* (Harris’ sparrow) and *Zonotrichia albicollis* (White-throated sparrow). Tissue sources include ethanol-preserved blood from live-caught and released specimens (53 individuals); preserved pectoral muscle, liver or heart tissue from specimens subsequently prepared as museum skins (23 individuals), and toe pad tissue from museum skins from critical sites (16 individuals, Table 1).

**Table 1:** **Details of the *Zonotrichia capensis *samples used in this study**

**Locality (Locality code)**^**a**^	**Lat./Long.**	**Sample type**^**b**^	**Sample ID**^**c**^	**CR**	**COI**	**ND2**	**16 s**	**CHD1Z**	**MELK**	**Fib5**
NW San Cristóbal, Chiapas, Mexico (1)	16°45’ N, 92°40’ W	SS	AMNH 766634	KC693420	-	-	-	-	-	-
Chichicastenango, Quiché, Guatemala (2)	14°56’ N, 91°07’ W	SS	AMNH 397960	KC693421	-	-	-	-	-	-
República Dominicana (3)	19°20’ N, 71°43’ W	BS	RD-ZCA	KC693437	-	-	-	-	-	-
			RD-ZCA-2	KC693436	-	-	-	-	-	-
			RD-ZCA-3	KC693439	-	-	-	-	-	-
			RD-ZCA-4	KC693438	-	-	-	-	-	-
			RD-ZCA-5	KC693440	-	-	-	-	-	-
La Georgina, San José, Costa Rica (4)	9°34’ N, 83°44’ W	MLHS	LSUMZ B16204	KC693418	KC693338	KC693306	KC693272	KC693466	KC693207	KC693240
			LSUMZ B16236	KC693419	KC693339	KC693307	KC693273	KC693467	KC693208	KC693241
Curaçao (5)	12°10' N, 69°00' W	SS	AMNH 174800	KC693428	-	-	-	-	-	-
			AMNH 174801	KC693429	-	-	-	-	-	-
El Junquito-Colonia Tovar, Aragua, Venezuela (6)	10°25' N, 67°13’ W	MLHS	GFB3125	KC693414	KC693346	KC693314	KC693280	KC693474	KC693215	-
			GFB3126	KC693415	KC693347	KC693315	KC693281	KC693475	KC693216	KC693248
			GFB3129	KC693413	KC693348	KC693316	KC693282	KC693476	KC693217	KC693249
			GFB3142	KC693417	KC693349	KC693317	KC693283	KC693477	KC693218	KC693250
			GFB3162	KC693416	KC693350	KC693318	KC693284	KC693478	KC693219	-
Laguna de La Herrera, Sabana de Bogotá, Colombia (7)	4°40’ N, 74°16’ W	SS	AMNH 803019	KC693430	-	-	-	-	-	-
			AMNH 803020	KC693431	-	-	-	-	-	-
			AMNH 803021	KC693432	-	-	-	-	-	-
Summit Mt. Roraima, Roraima, Venezuela (8)	5°14’ N, 60°47’ W	SS	AMNH 237128	KC693427	-	-	-	-	-	-
			AMNH 237131	KC693426	-	-	-	-	-	-
Quito, Pichincha, Ecuador (9)	0°13’ S, 78°30’ W	MLHS	PH-QUITO-2	KC693444	-	-	-	-	-	-
			PH-QUITO-4	KC693441	-	-	-	-	-	-
			PH-QUITO-6	KC693443	-	-	-	-	-	-
			PH-QUITO-7	KC693442	-	-	-	-	-	-
Rio Tocantins– Baião, Pará, Brazil (10)	2°50’ S, 49°36’ W	SS	AMNH 431444	KC693424	-	-	-	-	-	-
			AMNH 431447	KC693425	-	-	-	-	-	-
Huánuco-La Unión Rd., Huánuco, Perú (11)	9°53’ S, 76°32’ W	MLHS	LSUMZ B3603	KC693412	-	-	-	-	-	-
Cushi, Huánuco, Perú (12)	9°55’ S, 75°45’ W	MLHS	LSUMZ B8084	KC693409	KC693344	KC693312	KC693278	KC693472	KC693213	KC693246
			LSUMZ B8131	KC693410	KC693345	KC693313	KC693279	KC693473	KC693214	KC693247
Oxapampa, Pasco, Perú (13)	10°34’ S, 75°24’ W	MLHS	LSUMZ B1875	KC693411	KC693342	KC693310	KC693276	KC693470	KC693211	KC693244
Garanhuns, Pernambuco, Brazil (14)	8°54’ S, 36°29’ W	SS	AMNH 245037	KC693422	-	-	-	-	-	-
			AMNH 245038	KC693423	-	-	-	-	-	-
Chuspipita, La Paz, Bolivia (15)	16°18’ S, 67°47’ W	MLHS	LSUMZ B1277	KC693408	KC693335	KC693303	KC693269	KC693463	KC693204	KC693237
Catarata Arco Iris, Santa Cruz, Bolivia (16)	13°55’ S, 60°45’ W	MLHS	LSUMZ B14823	KC693406	KC693336	KC693304	KC693270	KC693464	KC693205	KC693238
			LSUMZ B14829	KC693403	KC693337	KC693305	KC693271	KC693465	KC693206	KC693239
Charagua, Santa Cruz, Bolivia (17)	19°48’ S, 61°50’ W	MLHS	LSUMZ B18743	KC693404	KC693340	KC693308	KC693274	KC693468	KC693209	KC693242
			LSUMZ B19108	KC693407	KC693343	KC693311	KC693277	KC693471	KC693212	KC693245
			LSUMZ B18745	KC693405	KC693341	KC693309	KC693275	KC693469	KC693210	KC693243
			LSUMZ B18751	KC693402	-	-	-	-	-	-
Yavi, Jujuy, Argentina (18)	22°8’ S, 65°28’ W	BS	SCL055	KC693359	-	-	-	-	-	-
			SCL056	KC693360	-	-	-	-	-	-
			SCL060	KC693361	-	-	-	-	-	-
			SCL062	KC693362	-	-	-	-	-	-
			SCL063	KC693363	KC693322	KC693288	KC693254	KC693448	KC693189	KC693223
			SCL066	KC693364	-	-	-	-	-	-
Tres Cruces, Jujuy, Argentina (19)	22°55’ S, 65°35’ W	BS	SCL071	KC693365	-	-	-	-	-	-
			SCL072	KC693366	-	-	-	-	-	-
			SCL074	KC693367	-	-	-	-	-	-
Iguazú, Misiones, Argentina (20)	25°34’ S, 54°34’ W	BS	SCL505	KC693396	-	-	-	-	-	-
Tafí del Valle, Tucumán, Argentina (21)	26°52’ S, 65°41’ W	BS	SCL084	KC693368	-	KC693289	KC693255	KC693449	KC693190	KC693224
			SCL085	KC693369	-	-	-	-	-	-
			SCL086	KC693370	-	-	-	-	-	-
			SCL088	KC693371	KC693323	KC693290	KC693256	KC693450	KC693191	KC693225
Rd. 42 30 k. SE of El Peñon, Catamarca, Argentina (22)	26°4’ S, 67°11’ W	MLHS	SCL009	KC693372	-	-	-	-	-	-
Quimilí, Santiago del Estero, Argentina (23)	27°38’ S, 62°25’ W	BS	SCL037	KC693353	-	-	-	-	-	-
			SCL038	KC693354	KC693321	KC693287	KC693253	KC693447	KC693188	KC693222
			SCL044	KC693355	-	-	-	-	-	-
			SCL048	KC693356	-	-	-	-	-	-
			SCL051	KC693357	-	-	-	-	-	-
			SCL052	KC693358	-	-	-	-	-	-
Reserva Natural del Iberá, Corrientes, Argentina (24)	28°06’ S, 57°06’ W	BS	SCL507	KC693397	KC693334	KC693302	KC693268	KC693462	KC693203	KC693236
			SCL508	KC693398	-	-	-	-	-	-
Mendoza, Mendoza, Argentina (25)	32°53’ S, 68°49’ W	BS	SCL502	KC693399	KC693332	KC693300	KC693266	KC693460	KC693201	-
			SCL503	KC693400	KC693333	KC693301	KC693267	KC693461	KC693202	KC693235
			SCL504	KC693401	-	-	-	-	-	-
La Reja, Buenos Aires, Argentina (26)	34°40 S’, 58°50’ W	BS	SCL010	KC693390	-	-	-	-	-	-
Magdalena, Buenos Aires, Argentina (27)	35°04’ S, 57°32’ W	BS	SCL110	KC693391	KC693328	KC693296	KC693262	KC693456	KC693197	KC693231
			SCL111	KC693392	-	-	-	-	-	-
			SCL112	KC693393	KC693329	KC693297	KC693263	KC693457	KC693198	KC693232
			SCL113	KC693394	KC693330	KC693298	KC693264	KC693458	KC693199	KC693233
			SCL115	KC693395	KC693331	KC693299	KC693265	KC693459	KC693200	KC693234
Isla de Mocha, Arauco, Chile (28)	38°23’ S, 73°52’ W	SS	AMNH 387432	KC693433	-	-	-	-	-	-
Puerto Montt, Llanquihue, Chile (29)	41°28’ S, 72°56’ W	SS	AMNH 182548	KC693435	-	-	-	-	-	-
Golfo de Ancud, Palena, Chile (30)	43°37’ S, 71°48’ W	SS	AMNH 182547	KC693434	-	-	-	-	-	-
Trelew, Chubut, Argentina (31)	43°15’ S, 65°18’ W	BS	SCL089	KC693373	-	-	-	-	-	-
			SCL090	KC693374	-	-	-	-	-	-
			SCL091	KC693375	-	-	-	-	-	-
			SCL092	KC693376	-	-	-	-	-	-
			SCL093	KC693377	-	-	-	-	-	-
Güer Aike, Santa Cruz, Argentina (32)	51°39’ S, 69°35’ W	BS	SCL096	KC693378	KC693324	KC693291	KC693257	KC693451	KC693192	KC693226
			SCL097	KC693379	-	-	-	-	-	-
			SCL098	KC693380	-	-	-	-	-	-
			SCL099	KC693381	-	-	-	-	-	-
			SCL100	KC693382	-	-	-	-	-	-
			SCL101	KC693383	-	-	-	-	-	-
			SCL102	KC693384	-	-	-	-	-	-
Ushuaia, Tierra del Fuego, Argentina (33)	54°48’ S, 68°18’ W	BS	SCL103	KC693385	-	-	-	-	-	-
			SCL104	KC693386	KC693325	KC693292	KC693258	KC693452	KC693193	KC693227
			SCL105	-	-	KC693293	KC693259	KC693453	KC693194	KC693228
			SCL106	KC693387	KC693326	KC693294	KC693260	KC693454	KC693195	KC693229
			SCL107	KC693388	KC693327	KC693295	KC693261	KC693455	KC693196	KC693230
			SCL108	KC693389	-	-	-	-	-	-

Localities from which *Zonotrichia capensis* individuals were sampled; genes amplified for each sample are indicated together with their GenBank accession numbers.

^a^*Zonotrichia albicollis* and *Zonotrichia querula* individuals obtained from Kingston, Ontario, Canada and Churchill, Manitoba, Canada, respectively were used as outgroups. GenBank accession Numbers: KC693445; KC693351; KC693319; KC693285; KC693479; KC693220; KC693251; KC693446; KC693352; KC693320; KC693286; KC693221; KC693252.

^b^MLHS, pectoral muscle, liver or heart sample; BS, blood sample; SS, museum study skin.

^c^AMNH, American Museum of Natural History; LSUMZ, Louisiana State University Museum of Zoology.

A portion of the mitochondrial control region (CR) was amplified from all individuals. We chose this locus for initial surveys because it has proved useful in similar studies of other birds [35]. We amplified and sequenced six additional markers from a subset of 32 individuals with representative divergent CR haplotypes (using fresh tissue) to help resolve phylogenetic structure. These sequences included three mitochondrial regions, cytochrome *c* oxidase subunit I (COI), 16 S rDNA (16 S) and NADH dehydrogenase subunit 2 (ND2); two Z-linked markers, chromodomain-helicase-DNA binding protein (CHD1Z) and maternal embryonic leucine zipper kinase (MELK); and one autosomal intron, intron 5 of the β-fibrinogen gene (Fib5).

Genomic DNA from fresh tissue was extracted using either a standard phenol chloroform protocol [36] or the DNeasy Tissue Kit (Qiagen, Mississauga, Canada), following the manufacturer’s instructions. DNA from toe pads was extracted using the latter method. For degraded DNA obtained from museum skins, we amplified a segment of the CR in small overlapping fragments generally shorter than 200 bp, using a series of primer pairs of our own design (see Additional file 1). For DNA from fresh tissue, an approximately 760 bp segment of the CR was amplified using primers ZnGluF3 and LCA1-REV271 (Additional file 1). PCR cocktails were prepared in a final volume of 25 μL KCl PCR buffer (Fermentas, Burlington, Canada) with the following composition: 10–20 ng of genomic DNA, 2.5 mM MgCl_2_, 0.5 μM of each primer, 0.5 mM of dNTPs, 1 U of Taq DNA polymerase (Fermentas). Negative controls were included for all PCRs. The thermocycling profiles were as follows: initial denaturation at 94°C for 2 min; 35 cycles with 30 s at 94°C, 20 s at designated annealing temperatures for each primer pair (Additional file 1), 30 s at 72°C; final extension at 72°C for 5 min. ND2, 16 S and MELK were amplified following PCR conditions outlined for CR, with the annealing temperatures and MgCl_2_ concentrations specified in the Additional file 1. COI amplification was conducted following Kerr *et al.*[37], while PCRs for CHD1Z and Fib5 followed Campagna *et al.*[38]. The small CR fragments amplified from toe pad DNA were electrophoresed in 0.5% agarose gels, and then excised and purified by the “freeze-squeeze” method [39]. The remaining PCR products were visualized on a 2% agarose gel using ethidium bromide and purified with the QIAquick PCR purification Kit (Qiagen). Most PCR products were sequenced bi-directionally (see Additional file 1 for primers) at the London Regional Genomics Centre (London, Canada). All sequences were deposited in Genbank; see Table 1.

We thus created two datasets: one of 92 ingroup plus 2 outgroup individuals with 396 bp of exclusively CR DNA sequences (hereafter, the CR dataset), and another of 32 ingroup plus 2 outgroup individuals with ≈4200 bp of seven molecular markers (hereafter, the multilocus dataset). Note that 92 of 93 individuals are represented in the CR dataset (see Table 1 for details).

### Genetic variability and phylogenetic analyses

Sequences were aligned using BIOEDIT v7.0.9.0 [40], and those coding for proteins were visually inspected to confirm lack of indels and translated into amino acids to verify absence of stop codons. The phylogeographic structure within *Z. capensis* was initially assessed by constructing a median-joining network using the CR dataset and NETWORK v4.6.1.0 (Fluxus Technology, Lt.). Differentiation between lineages identified by the network analysis was measured using Φ_ST_ values calculated with ARLEQUIN v3.5.1.2 [41] and average p distances using MEGA v5 [42]. For the former, significance was tested through 1000 random permutations. We also performed a Bayesian phylogenetic analysis using MRBAYES v3.1.2 [43,44], with the model of nucleotide evolution selected using JMODELTEST v0.1.1 [45]. The model that best fit the CR dataset according to the Akaike information criterion was the TrN [46] with a proportion of invariable sites (+I). The Bayesian analyses included two simultaneous runs of seven million generations using four incrementally heated Markov chains and default priors for all parameters. The analysis was run until the standard deviation of split frequencies was < 0.01, indicating convergence. We sampled trees every 100 generations, and after discarding the first 25% as burn-in, a 50% majority rule consensus was obtained from the combined posterior tree distribution of both runs. The potential scale reduction factor [47] was close to one for all parameters, implying that we had adequately sampled the posterior distributions. Finally, we used the ‘cumulative’ and ‘compare’ functions implemented in the software AWTY [48] to confirm that runs had reached stationarity.

We also conducted Bayesian phylogenetic analyses (as above) with the multilocus dataset using different partitioning strategies. We created trees using all seven loci, using mtDNA data alone (CR + COI + 16 S + ND2), using exclusively nuDNA (CHD1Z + MELK + Fib5), and using the DNA sequence data for each marker separately. For the seven-locus, concatenated dataset, we used two different strategies: 1. Specifying separate partitions for each gene and allowing them to vary independently according to the model of evolution selected by JMODELTEST. 2. Using a two-partition scheme, one for mitochondrial and one for nuclear loci, again each with separate models of evolution. Partitions were unlinked, estimating parameters separately while producing a posterior tree distribution from which a 50% majority rule consensus was obtained. To explore how different phylogenetic approaches might impact tree topology, we also performed Maximum Parsimony (MP) analyses using TNT v1.1 [49]. Heuristic searches consisted of 1000 random addition sequences with the TBR branch-swapping algorithm (retaining 100 trees per replication). A strict consensus was obtained from all resulting equally parsimonious trees. We assessed node robustness by performing 1000 standard bootstrap pseudoreplicates [50], each consisting of 100 random addition sequences followed by TBR (saving ten trees per pseudoreplicate).

### Estimations of diversification times

We estimated node ages using time to most recent common ancestor (TMRCA) with the Bayesian software BEAUti/BEAST v1.6.1 [51]. We calculated TMRCAs in absolute time using ND2 data with an approximate substitution rate consistent with the widely used clock calibration of ≈2% divergence per million years [52,53] (estimated for cytochrome *b* in birds). In a study on Hawaiian Honeycreepers, Lerner *et. al.*[54] estimated substitution rates for various genes, including cytochrome *b* and ND2, finding an average mitochondrial divergence rate of 1.8% per million years. Cytochrome *b* was found to diverge approximately 2.8% per million years, while estimations for ND2 were nearly double: 5.8% divergence per million years. Given that divergence rates could also differ between ND2 and cytochrome *b* in *Z. capensis*, as well as varying among lineages [55], we used a range of divergence values (1, 2, and 5% per million years) to analyze the sensitivity of time estimates to variations in clock calibrations. Analyses were run for 100 million generations using the nucleotide substitution models selected with JMODELTEST (GTR + I + G [56]). We used a relaxed uncorrelated lognormal clock and carried out calculations twice, once assuming constant population size and again using exponential growth. Because similar TMRCA estimates were obtained regardless of prior specified we only report the former. TRACER v1.5 [57] was used to assess convergence in parameter estimates by verifying that trends were not observed in traces of parameter values and that effective sample sizes exceeded 200.

### Population expansion tests and estimations of migration

We combined mitochondrial and nuclear data to explore the demographic history of *Z. capensis*. Mitochondrial data were derived either from the 92 CR sequences (396 bp) in the CR dataset or from the four mitochondrial loci from the 32 individuals in the multilocus dataset. In the latter case the four loci were concatenated (totaling ≈2700 bp) as they are physically linked in the mitochondrial genome. We inferred nuclear haplotypes for each locus (CHD1Z, MELK or Fib5) with DNASP v5.10 [58], using the data in subsequent analyses only if all sites had assignment probabilities ≥ 0.95. The three nuclear loci tested negative for recombination (p > 0.05) using the Phi test [59] implemented in SPLITSTREE v4 [60].

We tested for population demographic expansions or contractions by performing Fu’s Fs test [61] using ARLEQUIN and by calculating the exponential growth parameter *g* with LAMARC v2.1.8 [62]. Both Fu’s Fs test and the population growth parameter *g* were calculated independently for lineages A, B and C. Fu’s Fs test was conducted separately for the CR dataset, the combined mitochondrial data, and for inferred haplotypes from each of the CHD1Z, MELK and Fib5 datasets. Significance was assessed using 1000 simulated replicates and departures from neutrality were interpreted as consistent with population growth. The exponential population growth parameter *g* was calculated twice by placing either the CR dataset or the combined mitochondrial data and the inferred haplotypes from the three nuclear loci in four independent partitions. We ran LAMARC in maximum likelihood mode with the Felsenstein 84 [63] and GTR mutation models for nuclear and mitochondrial loci, respectively. Two simultaneous searches were conducted differing by 10% in the relative amount of heating incorporated. Each search consisted of two replicates of 20 initial and 5 final chains, saving 2000 and 10000 genealogies respectively in intervals of 20 generations.

**Figure 2:**
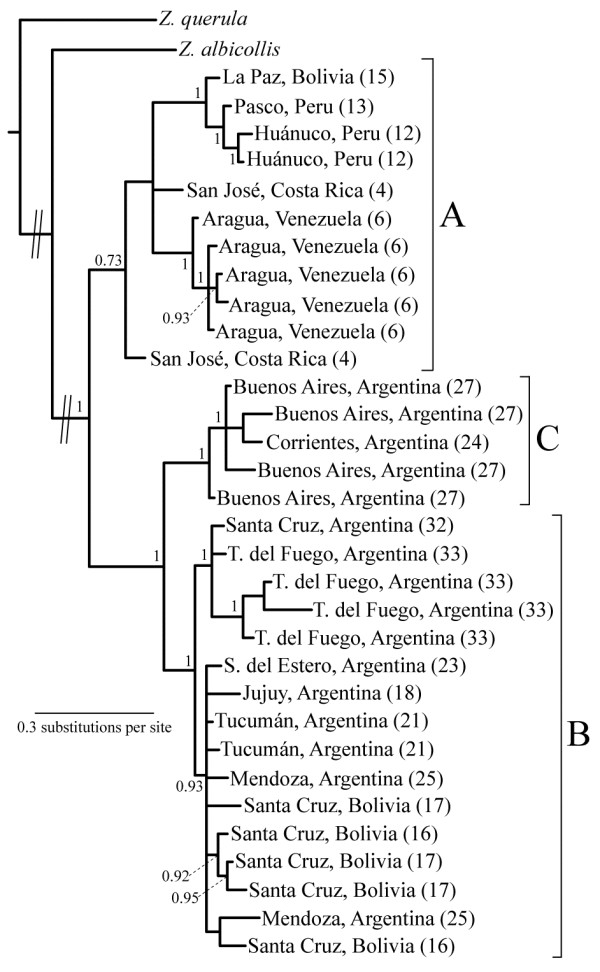
**Phylogenetic affinities of the three main *Z. capensis *lineages inferred using multilocus data.** Bayesian 50% majority rule consensus tree with posterior probabilities indicating node support derived using ≈4.2 kbp from the multilocus dataset. Each of the seven molecular markers was placed in a separate partition. Posterior probabilities below 0.90 were omitted for simplicity. Lineages **A**, **B** and **C** coincide with clades in the tree.

Finally, we estimated migration since lineages A, B and C split using the isolation with migration model implemented in the program IMa2 [64]. We used a three-population model; the relationship between lineages followed the topology shown in Figure 2. The program was run using the CR dataset and inferred haplotypes from the three nuclear loci and applying the HKY model [65] for each locus. We also estimated effective population sizes and splitting times, and simplified the model by estimating one migration rate parameter per population pair. Runs in M mode showed adequate mixing with 100 chains, the geometric heating model, and a burn-in period of 250 000 generations. IMa2 was run four times with different random seeds until at least ≈ 190 000 genealogies were saved. Joint-posterior density estimations of model parameters were obtained in L mode.

## Results

### Phylogeographic structure

The 92 individuals in the CR dataset contained 44 distinct haplotypes, differing at 1–13 sites (p distances from 0.25 to 3.28%). Average sequence divergence (p distances) between *Z. capensis* haplotypes and those of the two outgroups, *Z. albicollis* and *Z. querula*, was 6.61 ± 0.48% and 6.94 ± 0.45%, respectively. The median-joining network analysis (Figure 1B) identified three lineages (hereafter referred to as A, B and C) comprising haplotypes separated by at least two mutational steps. Pairwise p distance and Φ_ST_ between these three groups ranged from 1.48 to 2.51% and 0.66 to 0.74, respectively (Figure 1B). Lineage A includes haplotypes from northwestern South America (western Bolivia, Peru, Ecuador, Colombia and northern Venezuela), the Dominican Republic and Central America, including Chiapas, Mexico. Lineage B includes haplotypes from the Dominican Republic, Roraima (Venezuela), Santa Cruz (Bolivia), northwestern and central Argentina, Corrientes (Argentina), Chile and Patagonia (Argentina). Lineage C includes haplotypes from northeastern Argentina and eastern Brazil. Note that two localities show admixture of two lineages: haplotypes from A and B were found in the Dominican Republic (site 3, coded in orange in Figure 1A) and haplotypes from B and C were found in Corrientes, Argentina (site 24, coded in grey).

Figure 1C shows the Bayesian phylogeny from the CR dataset, where all *Z. capensis* individuals comprise a well-supported clade relative to congeneric outgroup taxa (posterior probability of 1.00). Among the three lineages identified by the network analysis, C was the only one that corresponded to a highly supported Bayesian clade (posterior probability of 0.99). Individuals from lineage B were embedded in a polytomy with clade C, while individuals from lineage A formed a polytomy that also included the clade composed of all haplotypes from lineages B and C (Figure 1C).

To improve resolution, we carried out analyses on the multilocus dataset (individuals marked with asterisks in Figure 1B and Figure 1C). The Bayesian phylogeny obtained by treating our seven loci in this dataset as separate partitions (Figure 2) is well resolved and most nodes are highly supported. The three lineages indentified in the network analysis correspond to clades in this tree, although support for clade A was low (posterior probability of 0.73). The topology shown in Figure 2 suggests clade A is sister to a clade comprising clades B and C. This analysis also found two subclades within clade B, one exclusive to Patagonia (Argentina) and the other deriving from central and northwestern Argentina and Santa Cruz (Bolivia). Alternative analyses suggest that this topology is robust, but mostly reflects information contained within the mitochondrial data (see below). Neither the Bayesian analysis that considered the mitochondrial and nuclear markers as separate partitions, nor the tree generated using maximum parsimony (Additional file 2) have strongly supported nodes contradicting those in Figure 2. Trees from the Bayesian analysis of mitochondrial data (placing each gene in a separate partition) and maximum parsimony of mitochondrial data resemble that of Figure 2, while trees derived solely from nuclear data were completely unstructured (Additional file 2; see Additional file 3 for individual gene trees), probably because of incomplete lineage sorting. Although we did not find fixed differences between lineages in the nuclear markers, there were significant haplotype frequency differences between lineage A and B (CHD1Z: F_ST_ = 0.056; MELK: F_ST_ = 0.141; Fib5: F_ST_ = 0.120).

### Demographic history

Lineage B was the only one showing strong evidence of population expansion consistently across the markers surveyed. With the exception of Fib5, the remaining loci each displayed negative and statistically significant Fu’s Fs values (Table 2). Moreover, the exponential growth parameter *g* was positive and statistically significant when calculated combining the CR dataset with the nuclear markers, providing further evidence of population growth. The signals of demographic expansions or contractions in lineages A and C were weak, with markedly smaller Fu’s Fs absolute values which were generally not statistically significant, and estimations of *g* with 95% confidence intervals that overlap zero (Table 2).

**Table 2:** **Tests for demographic expansions or contractions in the three *Zonotrichia capensis *lineages**

	**A**		**B**		**C**	
	**F**_**s**_	**p**	**F**_**s**_	**p**	**F**_**s**_	**p**
CR dataset	**−3.15 (25)**	**0.046**	**−25.23 (55)**	**<0.001**	**−3.77 (12)**	**0.006**
Combined mitochondrial	−1.68 (11)	0.150	**−5.74 (16)**	**0.003**	−1.48 (5)	0.096
CHD1Z	**−2.68 (20)**	**0.006**	**−1.79 (28)**	**0.036**	−0.182 (8)	0.191
MELK	6.61 (18)	0.989	**−2.188 (30)**	**0.043**	−0.427 (6)	0.180
Fib5	2.51 (16)	0.900	0.67 (24)	0.640	3.70 (6)	0.950
*g* (CR dataset + nuDNA)	165.77^ns^ (79)		669.98^*^ (137)		−30.74^ns^ (32)	
*g* (combined mitochondrial + nuDNA)	−47.43^ns^ (65)		124.83^ns^ (98)		452.67^ns^ (25)	

Results from Fu’s Fs test and calculations of the exponential growth parameter *g* for *Zonotrichia capensis* lineages A, B and C (number of sequences used in each case indicated in parenthesis). Fu’s Fs test was performed on mitochondrial loci from the CR dataset, the combined mitochondrial data and for inferred haplotypes from nuclear markers. Statistically significant p values (< 0.05) are indicated in bold. For *g*, statistical significance was assessed through confidence intervals (see text for details).

*99% confidence interval does not include 0.

ns, not statistically significant (95% confidence interval includes 0).

The events that generated the three clades most likely occurred during the Pleistocene (*ca.* 0.01 to 2.6 million years ago). The deepest split, the node that separates clade A from B and C in Figure 2, has a mean TMRCA of 1.45 million years assuming 2% divergence per million years (Table 3). If the limits of the 95% high posterior density intervals are considered, the former splitting time overlaps with that separating clades B and C. Moreover, if we consider alternative “slow” (1% divergence per million years) or “fast” (5% divergence per million years) molecular clock calibrations, these estimates also overlap with the split within clade B (Figure 2, Table 3). These results imply that the major cladogenic events in *Z. capensis* occurred close in time, an assertion that is also suggested by the short internode distances in Figure 2 and the extensive overlap in the posterior probability curves from splitting times estimated using IMa2 (Additional file 4). Finally, estimates of migration between pairs of lineages had 95% high posterior density intervals that overlapped with zero (Additional file 4).

**Table 3:** **Estimations of timing of splitting events between *Zonotrichia capensis *lineages**

	**Mean “fast” clock (5%)**	**Low 95% HPD (2%)**	**Mean (2%)**	**High 95% HPD (2%)**	**Mean “slow” clock (1%)**
ABC (32)	0.58	0.70	**1.45**	2.38	2.88
BC (21)	0.34	0.40	**0.86**	1.42	1.71
B (16)	0.15	0.15	**0.37**	0.64	0.74

TMRCA and 95% confidence intervals (in units of millions of years) for three different nodes in the tree shown in Figure 2. Values were estimated using BEAST and ND2 sequences with a ≈2% per million year divergence rate [52] (in bold). Sample sizes are in parenthesis after each node. The mean values obtained using alternative calibrations of 1 and 5% per million years (“slow” and “fast” clocks, respectively) are also reported.

## Discussion

Our analyses of sequence data from samples obtained across the range of *Z. capensis* revealed three major lineages within the species: a Middle American and northwestern South American lineage that also includes the Dominican Republic (A); a lineage encompassing the Dominican Republic, Roraima (Venezuela) and a large swath from La Paz (Bolivia) south to Tierra del Fuego, Argentina (B); and a lineage spanning the eastern portion of the species range (C: eastern Argentina and Brazil). Phylogenetic analyses suggest that clade A is sister to the other lineages. The events that gave rise to these lineages most likely occurred during the Pleistocene and were near-coincident in time. Demographic analyses implied no gene flow among lineages during their diversification; hence localities with individuals from more than one lineage in the Dominican Republic and Corrientes (Argentina) may represent areas of repeated colonization or of secondary contact (Figure 1) and should be the focus of future studies with more in depth sampling. Finally, the most widely distributed lineage shows the strongest signal of population expansion. It is unlikely that the three *Z. capensis* lineages that span the majority of South America are panmictic and there is evidence of shallow phylogeographic structure within A and B (Figure 2), although different ways of analyzing the data do not always recover clades within these lineages (see Additional files 2 and 3). IMa2 assumes lack of population structure within lineages; thus we must be careful to not over-interpret our demographic results. While Strasburg and Rieseberg [66] found the algorithms employed by IMa2 are robust to violations of the assumption of panmixia, future studies with finer geographic resolution should seek to validate our current findings. Below we discuss these results in the context of our motivating questions.

### Phylogeographic patterns

The deepest split within the species is between lineage A (Middle and northwestern South America) and all other South American samples (B and C). This pattern is consonant with previous findings in other Neotropical avian taxa (e.g., [67-69]), and has been generally interpreted as the product of vicariant events on widespread ancestral populations mediated by the uplift of the Andes Mountains. Since splits between *Z. capensis* lineages date roughly to the Pleistocene, when Andean uplift was essentially complete [12], and given that the species mostly inhabits open country, which in tropical latitudes is restricted to higher elevations [18,70], this explanation seems implausible. Alternatively, Quaternary climatic processes have also deeply influenced speciation in Neotropical taxa (e.g., [17,71]), particularly through climate changes related to glacial cycles and their effects on species distributions. Thus Pleistocene glaciations may have helped isolate and shape *Z. capensis* lineages. The pattern could also reflect a history of geographical isolation and colonization of South America largely consistent with Chapman’s model [18] - see below.

It is possible that the genus originated in North America with subsequent differentiation of the four northern species and a southward expansion of the *Z. capensis* ancestor that led to the genealogical patterns evident in the species. We note that the divergence between our two outgroup taxa (≈1.8% p distance in CR between *Z. albicollis* and *Z. querula*) is comparable to that among *Z*. *capensis* lineages, implying a similar timeframe for diversification between north-temperate *Zonotrichia* species and lineages within the single Neotropical species. Other studies of lower latitude taxa have shown greater neutral genetic divergence and stronger phylogeographic structure than their temperate counterparts (e.g., [4,5,72]).

Our results are consistent with two South American colonization hypotheses that assume a Central America origin for *Z. capensis* and range expansion facilitated by open country. One is consistent with Chapman’s [18] inference: An eastward colonization along the margins of the Caribbean and Atlantic Ocean and then southwards into Brazil, together with a colonization southwards along the Andean chain towards Argentina. The second involves this same southward Andean colonization, followed by an eastward expansion into low elevation open habitats south of the southern limits of the Amazonian forest systems of the eastern Andean slopes (currently at ≈27°S). This expansion would have continued southward into Patagonia and northeast along the “arid diagonal” of the chaco-cerrado-caatinga into eastern Argentina, Paraguay and sub-Amazonian Brazil. Additional sampling from areas that are poorly represented in or entirely absent from our study (e.g., southern Venezuela, Guyana, French Guyana, Suriname, Brazil, northern Chile and western Bolivia) will allow greater insight into how this species colonized South America.

In earlier work, Lougheed and Handford [31] and Lougheed *et al.*[32] speculated that the genetic differentiation uncovered in northwestern Andean Argentina represented a secondary contact of two postulated primary clades representing the major routes of colonization of South America proposed by Chapman [18]. Our results show the differentiation that they found is encompassed within lineage B and perhaps a consequence of more recent distributional changes.

Finally, while we found substantial phylogeographic structure within *Z. capensis*, lineages in this species are not as divergent as those in other Neotropical species with comparable ranges (e.g., *Troglodytes aedon*: [73]; *Cistothorus platensis*: M. Robbins, University of Kansas Biodiversity Institute, personal communication). Recent mitochondrial surveys including many Neotropical bird taxa have also shown deep divergence within species with much smaller geographic ranges (e.g., see [37,74]). While future studies should clarify if these deeply diverged lineages in other taxa are reproductively isolated cryptic species, we suspect that the ecological flexibility of the rufous-collared sparrow, which is known to rapidly colonize newly-opened terrain, explains the lack of deep divergence over a wide range spanning over 70 degrees of latitude.

### Lack of correlation between phylogeographic structure and phenotypic variation

Mitochondrial phylogeographic structure does not reflect the subspecific taxonomy of the rufous-collared sparrow [75], consonant with the findings of other studies in many taxa (e.g., [76-78]). Moreover, Handford [24] showed that *Z. capensis* subspecies cannot be recovered even from morphometric data. This is perhaps not surprising given that phenotypic responses to local environmental circumstances may occur rapidly regardless of historical fragmentation of populations (e.g., [79,80]). Furthermore, no studies have yet been published quantifying heritability in the traits that differentiate *Z. capensis* subspecies.

The three diagnosed lineages each include several subspecies (at least seven in lineage A, eight in lineage B, and three in lineage C – see [18] for a detailed discussion on subspecies distributions). Moreover, some subspecies are represented in two lineages (e.g., *Z. c. antillarum* in lineages A and B; *Z. c. hypoleuca* in lineages B and C). However one well-supported subclade within lineage B included individuals exclusively from the southernmost portion of the species range, all belonging to subspecies *Z. c. australis* (Figure 2). This suggests that glaciations in Patagonia might have played a role in isolating southern populations of rufous-collared sparrows, which subsequently expanded their range after the ice retreated, a scenario that has been documented in other southern Neotropical taxa [7].

Our findings provide no clear evidence that variation in vocal behavior in *Z. capensis* relates to major phylogeographic divisions. Habitat-related geographical vocal dialects are documented from Argentina in both lineages B and C, with high levels of song differentiation found throughout northwestern and central Argentina, which is occupied by the single lineage B, and similarly differentiated songs in grassland and wooded environments in the parts of Argentina occupied by lineage C (Entre Rios, Corrientes, Buenos Aires). Habitat-related dialects extend at least into northern Patagonia, with distinctive songs in the Patagonian shrub-steppe and Andean woodlands, but we must acknowledge that vocal behaviour is poorly understood south of ≈40°S in the range occupied by highly migratory populations. Similarly, though we know that there is geographical variation in song in Brazil [81], it is too poorly known for generalizations to be made.

On the other hand, substantial individual repertoires and an evident lack of habitat-related dialects are known only from regions encompassed by lineage A (Costa Rica; Ecuador). This raises the possibility that dialects are the derived condition (and that the systems of song dialects that exist in some of the North American *Zonotrichia* are independently derived). In any event, the association between vegetation structure and vocal dialects must have developed recently and dialect distribution is not reflected in the genealogical relationships that we have reported on here. Thus, we provisionally conclude that vocal variation in rufous-collared sparrows is probably a manifestation of cultural evolution that overlies deeper intraspecific genealogical patterns.

## Conclusions

These results suggest a Pleistocene history of colonization and population expansion in the rufous-collared sparrow from a probable Central American origin. Diversification occurred during the Pleistocene, a time of substantial global climate fluctuations and variation in the extent and distribution of different vegetation types. The impressive diversity in morphology, migratory habit, and vocal system of this species most probably represents recent responses to local conditions and overlies deeper patterns of lineage diversity that are products of geographical isolation and the colonization history of South America. That subspecies are not reflected in major phylogeographic divisions is not surprising in light of patterns exhibited by many other taxa, but this does not preclude the possibility that local adaptation may modulate the evolutionary trajectories of this species.

The deepest split in the species is between a Central American/tropical Andean clade and all other populations to the south and east. Phylogeographic patterns are consistent with two colonization scenarios of South America following either: a) a colonization southwards along the Andes and an eastward and then southward colonization along the margins of the Caribbean and Atlantic Ocean, or b) this same southward Andean colonization, followed by an eastward and then southward colonizations into Patagonia and towards the northeast into eastern Argentina, Paraguay and Brazil. To enhance insights into the processes that influenced the evolutionary history of this widespread passerine, future investigations should increase geographic sampling intensity in the northeastern portion of the species range and evaluate alternative scenarios through a more rigorous hypothesis-testing framework (such as Approximate Bayesian computation).

## Competing interests

The authors declare that they have no competing interests.

## Authors’ contributions

SCL, PLT and PH conceived the ideas; SCL, LC, JAD and DAL collected the data; SCL and LC analyzed the data and led the writing, with help from the other authors. All authors read and approved the final manuscript.

## Authors’ information

Collectively, we are interested in understanding the origins of biodiversity from the level of local adaptation and limiting gene flow in single landscapes, through the genetics of entire species’ ranges, to understanding the causes of diversification of entire clades. Our research spans the Americas, with particular emphasis on phylogeography and phylogenetics of select anurans, squamates and birds.

## Supplementary Material

Additional file 1 Table of all primers and PCR conditions used to amplify loci in the study. Click here for file

Additional file 2 Bayesian and Maximum Parsimony trees generated using alternative partitioning strategies to analyze the multilocus dataset. Click here for file

Additional file 3 Bayesian individual gene trees. Click here for file

Additional file 4 **Posterior density curves for splitting times, migration rates and effective population sizes estimated using IMa2.** (PDF 236 kb) Click here for file
