# Supplementary material for: Continental phylogeography of an ecologically and morphologically diverse Neotropical songbird, Zonotrichia capensis
Source: BMC Evol Biol. 2013 Mar 1;13:58. doi: 10.1186/1471-2148-13-58 (PMC3632491; doi:10.1186/1471-2148-13-58)
Supplement: Additional file 1 — Table of all primers and PCR conditions used to amplify loci in the study. [file 1471-2148-13-58-S1.pdf]

Additional File 1

Primers and PCR conditions used to amplify loci in this study.

Locus	Primer name and sequence	PCR product size	Annealing temperature	Mg^{2+} concentration	Reference
CR	ZnGluF3 5’-GCCTCTCTCCGAGATTTACGGC-3’<br>LCA1-REV271 5’-CTGTGAAGAGCAAGGAGAG-3’	760 bp	63ºC	2.5 mM	This study
CR	LCA2-FOR302 5’-CATACGCTATGTCTCTCCAC-3’<br>LCA2-REV455 5’-GTCTYTGGGTGACGCTTG-3’	149 bp	53ºC	2.5 mM	This study
CR	LCA2-FOR403 5’-CCAAGTGTTCCTACCYARG-3’<br>LCA2-REV506 5’-GGATCTTCCTCGTTCACTAG-3’	102 bp	53ºC	2.5 mM	This study
CR	LCA2-FOR459 5’-GRRACTTATCTGYTATRCAC-3’<br>LCA1-REV188 5’-GAGTGCTTGAAGGCTGTTG-3’	131 bp	53ºC	2.5 mM	This study
CR	LCA2-FOR486 5’-CTAGTGAACGAGGAAGATCC-3’<br>LCA1-REV120 5’-CGATCAATAGATAMCCATG-3’	133 bp	53ºC	2.5 mM	This study
CR	LCA1-FOR70 5’-CAACAGCCTTCAAGCACTCC-3’<br>LCA1-REV228 5’-GAAGTTACAACCAATAGCG-3’	168 bp	53ºC	2.5 mM	This study
CR	LCA1-FOR162 5’-TCGGTTATTGGCCTTGAGC-3’<br>LCA1-REV271 5’-CTGTGAAGAGCAAGGAGAG-3’	107 bp	53ºC	2.5 mM	This study
CR	LCA2-FOR302 5’-CATACGCTATGTCTCTCCAC-3’<br>LCA1-REV271 5’-CTGTGAAGAGCAAGGAGAG-3’	378 bp	61ºC	2.5 mM	This study
16s rDNA	16SbrH 5’-CCGGTCTGAACTCAGATCACGT-3’<br>16SarL 5’-CGCCTGTTTATCAAAAACAT-3’	571 bp	58ºC	3 mM	[1]
COI	BirdF1 5’-TTCTCCAACCACAAAGACATTGGCAC-3’<br>COIbirdR2 5’-ACGTGGGAGATAATTCCAAATCCTGG-3’	672 bp	51ºC	2.5 mM	[2]
ND2	H6313 5’-CTCTTATTTAAGGCTTTGAAGGC-3’	989 bp	54ºC	2 mM	[3]

	L5216 5’-GCCCATACCCCRAMAATG-3’				
CHD1Z	2550F 5’-GTTACTGATTCGTCTACGAGA-3’	611 bp	58ºC	2.5 mM	[4]
	2718R 5’-ATTGAAATGATCCAGTGCTTG-3’				
MELK	For 5’-CAACTTTTTCACCAGTAAG-3’	345 bp	55ºC	2 mM	[5]
	Rev 5’-CTACGAGGAAATTCTCAA-3’				
Fib5	Fib5 5’- CGCCATACAGAGTATACTGTGACAT-3’	562 bp	54ºC	2.5 mM	[6]
	Fib6 5’-GCCATCCTGGCGATTCTGAA-3’				

1. Palumbi S, Martin A, Romano S, McMillan WO, Stice L, Grabowski G: *The simple fool’s guide to PCR, version 2*. Honolulu: University of Hawaii; 1991.

2. Kerr KC, Lijtmaer DA, Barreira AS, Hebert PDN, Tubaro PL: **Probing evolutionary patterns in Neotropical birds through DNA barcodes.** *PloS One* 2009, **4:**e4379.

3. Sorenson MD, Ast JC, Dimcheff DE, Yuri T, Mindell DP: **Primers for a PCR-based approach to mitochondrial genome sequencing in birds and other vertebrates.** *Mol Phylogenet Evol* 1999, **12:**105-114.

4. Fridolfsson AK, Ellegren H: **A simple and universal method for molecular sexing of non-ratite birds.** *J Avian Biol* 1999, **30:**116-121.

5. Backström N, Lindell J, Zhang Y, Palkopoulou E, Qvarnström A, Saetre G-P, Ellegren H: **A high-density scan of the Z chromosome in *Ficedula* flycatchers reveals candidate loci for diversifying selection.** *Evolution* 2010, **64:**3461-3475.

6. Kimball RT, Braun EL, Barker FK, Bowie RCK, Braun MJ, Chojnowski JL, Hackett SJ, Han KL, Harshman J, Heimer-Torres V, Holznagel W, Huddleston CJ, Marks BD, Miglia KJ, Moore WS, Reddy S, Sheldon FH, Smith JV, Witt CC, Yuri T: **A well-tested set of primers to amplify regions spread across the avian genome.** *Mol Phylogenet Evol* 2009, **50:**654-660.
