# Supplementary material for: Continental phylogeography of an ecologically and morphologically diverse Neotropical songbird, Zonotrichia capensis
Source: BMC Evol Biol. 2013 Mar 1;13:58. doi: 10.1186/1471-2148-13-58 (PMC3632491; doi:10.1186/1471-2148-13-58)
Supplement: Additional file 2 — Bayesian and Maximum Parsimony trees generated using alternative partitioning strategies to analyze the multilocus dataset. [file 1471-2148-13-58-S2.pdf]

Additional File 2

Bayesian and Maximum Parsimony (MP) trees generated using alternative partitioning strategies to analyze the multilocus dataset. Bayesian topologies are 50% majority rule consensus trees with posterior probabilities indicating node support. MP trees represent a strict consensus from equally parsimonious topologies with node support assessed through 1000 standard bootstrap pseudoreplicates. When phylogenetic resolution was sufficient, clades corresponding to lineages A to C are indicated. Posterior probabilities below 0.90 were omitted for simplicity. (a) Bayesian topology obtained by placing mitochondrial and nuclear data in different partitions. Bayesian trees produced partitioning mitochondrial (b) or nuclear (c) data alone by gene. MP topologies obtained using the entire multilocus dataset (d). MP trees built with mitochondrial (e) or nuclear (f) data alone.

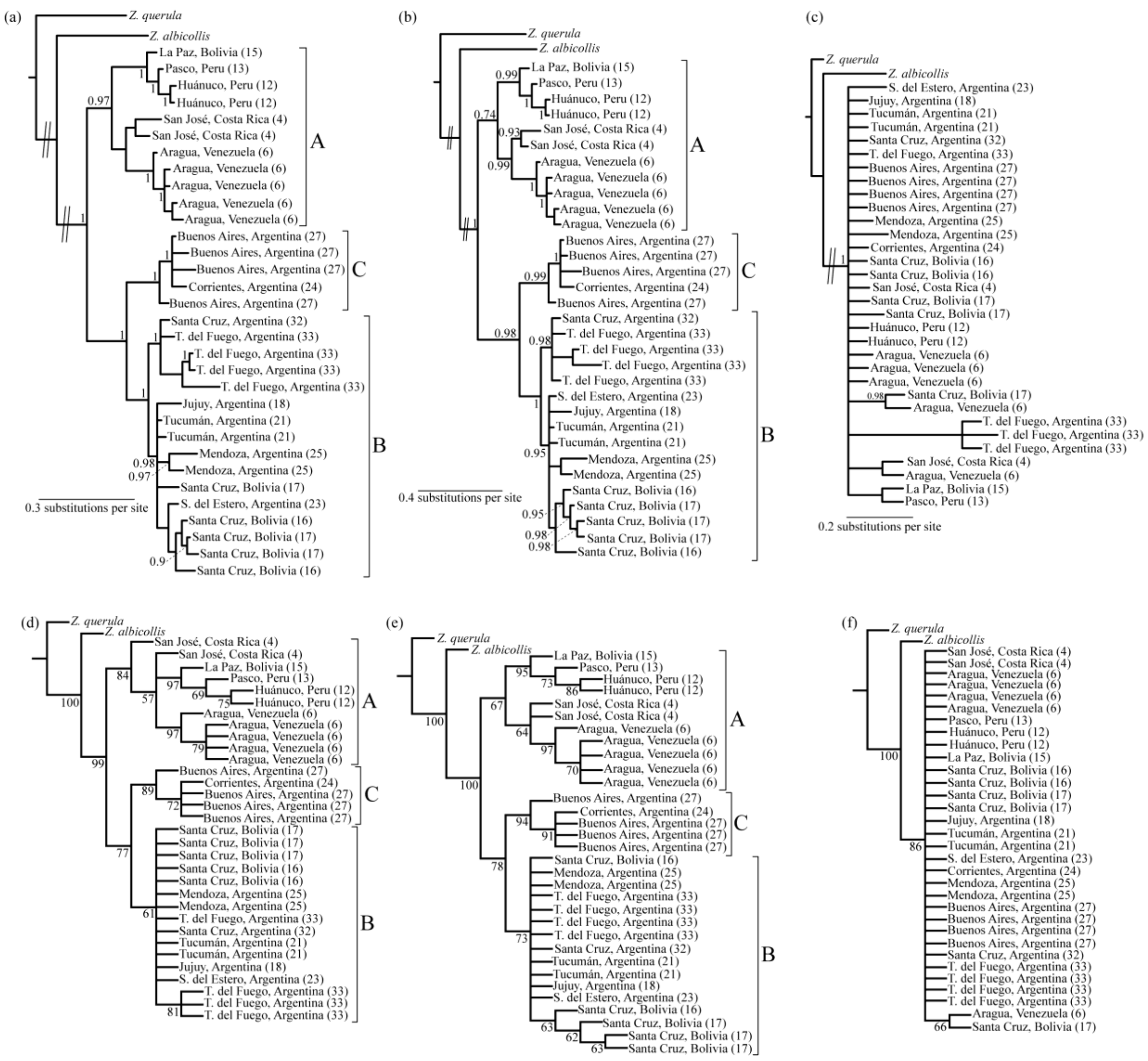
(a)
Z. querula
Z. albicollis
La Paz, Bolivia (15)
Pasco, Peru (13)
Huánuco, Peru (12)
Huánuco, Peru (12)
San José, Costa Rica (4)
San José, Costa Rica (4)
Aragua, Venezuela (6)
Aragua, Venezuela (6)
Aragua, Venezuela (6)
Aragua, Venezuela (6)
Aragua, Venezuela (6)
A
Buenos Aires, Argentina (27)
Buenos Aires, Argentina (27)
Buenos Aires, Argentina (27)
Corrientes, Argentina (24)
Buenos Aires, Argentina (27)
C
Santa Cruz, Argentina (32)
T. del Fuego, Argentina (33)
T. del Fuego, Argentina (33)
T. del Fuego, Argentina (33)
T. del Fuego, Argentina (33)
Jujuy, Argentina (18)
Tucumán, Argentina (21)
Tucumán, Argentina (21)
Mendoza, Argentina (25)
Mendoza, Argentina (25)
Santa Cruz, Bolivia (17)
S. del Estero, Argentina (23)
Santa Cruz, Bolivia (16)
Santa Cruz, Bolivia (17)
Santa Cruz, Bolivia (17)
Santa Cruz, Bolivia (16)
B
0.3 substitutions per site
(b)
Z. querula
Z. albicollis
La Paz, Bolivia (15)
Pasco, Peru (13)
Huánuco, Peru (12)
Huánuco, Peru (12)
San José, Costa Rica (4)
San José, Costa Rica (4)
Aragua, Venezuela (6)
Aragua, Venezuela (6)
Aragua, Venezuela (6)
Aragua, Venezuela (6)
Aragua, Venezuela (6)
A
Buenos Aires, Argentina (27)
Buenos Aires, Argentina (27)
Buenos Aires, Argentina (27)
Corrientes, Argentina (24)
Buenos Aires, Argentina (27)
C
Santa Cruz, Argentina (32)
T. del Fuego, Argentina (33)
T. del Fuego, Argentina (33)
T. del Fuego, Argentina (33)
T. del Fuego, Argentina (33)
S. del Estero, Argentina (23)
Jujuy, Argentina (18)
Tucumán, Argentina (21)
Tucumán, Argentina (21)
Mendoza, Argentina (25)
Mendoza, Argentina (25)
Santa Cruz, Bolivia (16)
Santa Cruz, Bolivia (17)
Santa Cruz, Bolivia (17)
Santa Cruz, Bolivia (17)
Santa Cruz, Bolivia (16)
B
0.4 substitutions per site
(c)
Z. querula
Z. albicollis
S. del Estero, Argentina (23)
Jujuy, Argentina (18)
Tucumán, Argentina (21)
Tucumán, Argentina (21)
Santa Cruz, Argentina (32)
T. del Fuego, Argentina (33)
Buenos Aires, Argentina (27)
Buenos Aires, Argentina (27)
Buenos Aires, Argentina (27)
Buenos Aires, Argentina (27)
Mendoza, Argentina (25)
Mendoza, Argentina (25)
Corrientes, Argentina (24)
Santa Cruz, Bolivia (16)
Santa Cruz, Bolivia (16)
San José, Costa Rica (4)
Santa Cruz, Bolivia (17)
Santa Cruz, Bolivia (17)
Huánuco, Peru (12)
Huánuco, Peru (12)
Aragua, Venezuela (6)
Aragua, Venezuela (6)
Aragua, Venezuela (6)
Santa Cruz, Bolivia (17)
Aragua, Venezuela (6)
T. del Fuego, Argentina (33)
T. del Fuego, Argentina (33)
T. del Fuego, Argentina (33)
San José, Costa Rica (4)
Aragua, Venezuela (6)
La Paz, Bolivia (15)
Pasco, Peru (13)
0.2 substitutions per site
(d)
Z. querula
Z. albicollis
San José, Costa Rica (4)
San José, Costa Rica (4)
La Paz, Bolivia (15)
Pasco, Peru (13)
Huánuco, Peru (12)
Huánuco, Peru (12)
Aragua, Venezuela (6)
Aragua, Venezuela (6)
Aragua, Venezuela (6)
Aragua, Venezuela (6)
Aragua, Venezuela (6)
A
Buenos Aires, Argentina (27)
Corrientes, Argentina (24)
Buenos Aires, Argentina (27)
Buenos Aires, Argentina (27)
Buenos Aires, Argentina (27)
C
Santa Cruz, Bolivia (17)
Santa Cruz, Bolivia (17)
Santa Cruz, Bolivia (17)
Santa Cruz, Bolivia (16)
Santa Cruz, Bolivia (16)
Mendoza, Argentina (25)
Mendoza, Argentina (25)
T. del Fuego, Argentina (33)
Santa Cruz, Argentina (32)
Tucumán, Argentina (21)
Tucumán, Argentina (21)
Jujuy, Argentina (18)
S. del Estero, Argentina (23)
T. del Fuego, Argentina (33)
T. del Fuego, Argentina (33)
T. del Fuego, Argentina (33)
B
(e)
Z. querula
Z. albicollis
La Paz, Bolivia (15)
Pasco, Peru (13)
Huánuco, Peru (12)
Huánuco, Peru (12)
San José, Costa Rica (4)
San José, Costa Rica (4)
Aragua, Venezuela (6)
Aragua, Venezuela (6)
Aragua, Venezuela (6)
Aragua, Venezuela (6)
Aragua, Venezuela (6)
A
Buenos Aires, Argentina (27)
Corrientes, Argentina (24)
Buenos Aires, Argentina (27)
Buenos Aires, Argentina (27)
Buenos Aires, Argentina (27)
C
Santa Cruz, Bolivia (16)
Mendoza, Argentina (25)
Mendoza, Argentina (25)
T. del Fuego, Argentina (33)
T. del Fuego, Argentina (33)
T. del Fuego, Argentina (33)
T. del Fuego, Argentina (33)
Santa Cruz, Argentina (32)
Tucumán, Argentina (21)
Tucumán, Argentina (21)
Jujuy, Argentina (18)
S. del Estero, Argentina (23)
Santa Cruz, Bolivia (16)
Santa Cruz, Bolivia (17)
Santa Cruz, Bolivia (17)
Santa Cruz, Bolivia (17)
B
(f)
Z. querula
Z. albicollis
San José, Costa Rica (4)
San José, Costa Rica (4)
Aragua, Venezuela (6)
Aragua, Venezuela (6)
Aragua, Venezuela (6)
Aragua, Venezuela (6)
Pasco, Peru (13)
Huánuco, Peru (12)
Huánuco, Peru (12)
La Paz, Bolivia (15)
Santa Cruz, Bolivia (16)
Santa Cruz, Bolivia (16)
Santa Cruz, Bolivia (17)
Santa Cruz, Bolivia (17)
Jujuy, Argentina (18)
Tucumán, Argentina (21)
Tucumán, Argentina (21)
S. del Estero, Argentina (23)
Corrientes, Argentina (24)
Mendoza, Argentina (25)
Mendoza, Argentina (25)
Buenos Aires, Argentina (27)
Buenos Aires, Argentina (27)
Buenos Aires, Argentina (27)
Buenos Aires, Argentina (27)
Santa Cruz, Argentina (32)
T. del Fuego, Argentina (33)
T. del Fuego, Argentina (33)
T. del Fuego, Argentina (33)
T. del Fuego, Argentina (33)
Aragua, Venezuela (6)
Santa Cruz, Bolivia (17)
