# Supplementary material for: Continental phylogeography of an ecologically and morphologically diverse Neotropical songbird, Zonotrichia capensis
Source: BMC Evol Biol. 2013 Mar 1;13:58. doi: 10.1186/1471-2148-13-58 (PMC3632491; doi:10.1186/1471-2148-13-58)
Supplement: Additional file 3 — Bayesian individual gene trees. [file 1471-2148-13-58-S3.pdf]

Additional File 3

Bayesian trees generated using individual genes from the multilocus dataset. Bayesian topologies are 50% majority rule consensus trees with posterior probabilities indicating node support. Samples belonging to lineages A, B and C are indicated despite them not conforming clades in some trees. Genes and models of nucleotide evolution are as follows: (a) COI, HKY+I; (b) CR, GTR+I; (c) ND2, GTR+I+G; (d) 16s, GTR+I+G; (e) MELK, GTR+I; (f) CHD1Z, HKY+G; (g) Fib5, GTR+I+G. Trees obtained from nuclear data were completely unstructured, thus the topology from Figure 2 is mainly derived from mtDNA. Although resolution varies between mitochondrial genes, there are no highly supported nodes that contradict the topology from Figure 2.

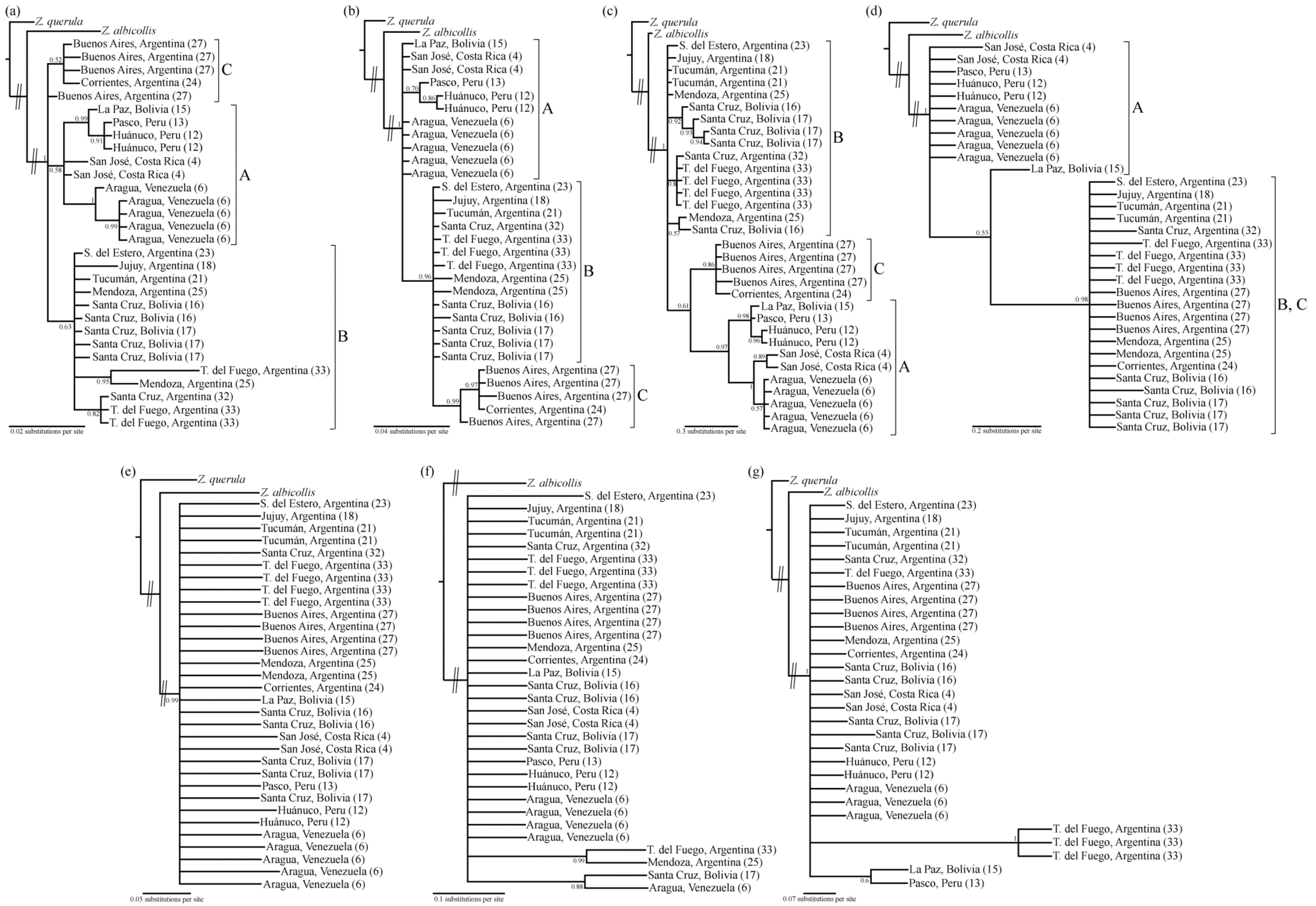

(a)
Z. querula
Z. albicollis
Buenos Aires, Argentina (27)
Buenos Aires, Argentina (27)
Buenos Aires, Argentina (27)
Corrientes, Argentina (24)
Buenos Aires, Argentina (27)
C
La Paz, Bolivia (15)
Pasco, Peru (13)
Huánuco, Peru (12)
Huánuco, Peru (12)
San José, Costa Rica (4)
San José, Costa Rica (4)
Aragua, Venezuela (6)
Aragua, Venezuela (6)
Aragua, Venezuela (6)
Aragua, Venezuela (6)
Aragua, Venezuela (6)
A
S. del Estero, Argentina (23)
Jujuy, Argentina (18)
Tucumán, Argentina (21)
Mendoza, Argentina (25)
Santa Cruz, Bolivia (16)
Santa Cruz, Bolivia (16)
Santa Cruz, Bolivia (17)
Santa Cruz, Bolivia (17)
Santa Cruz, Bolivia (17)
T. del Fuego, Argentina (33)
Mendoza, Argentina (25)
Santa Cruz, Argentina (32)
T. del Fuego, Argentina (33)
T. del Fuego, Argentina (33)
B
0.52
0.99
0.91
1
0.58
1
0.99
0.63
0.95
0.82
0.02 substitutions per site
(b)
Z. querula
Z. albicollis
La Paz, Bolivia (15)
San José, Costa Rica (4)
San José, Costa Rica (4)
Pasco, Peru (13)
Huánuco, Peru (12)
Huánuco, Peru (12)
Aragua, Venezuela (6)
Aragua, Venezuela (6)
Aragua, Venezuela (6)
Aragua, Venezuela (6)
Aragua, Venezuela (6)
A
S. del Estero, Argentina (23)
Jujuy, Argentina (18)
Tucumán, Argentina (21)
Santa Cruz, Argentina (32)
T. del Fuego, Argentina (33)
T. del Fuego, Argentina (33)
T. del Fuego, Argentina (33)
Mendoza, Argentina (25)
Mendoza, Argentina (25)
Santa Cruz, Bolivia (16)
Santa Cruz, Bolivia (16)
Santa Cruz, Bolivia (17)
Santa Cruz, Bolivia (17)
Santa Cruz, Bolivia (17)
B
Buenos Aires, Argentina (27)
Buenos Aires, Argentina (27)
Buenos Aires, Argentina (27)
Corrientes, Argentina (24)
Buenos Aires, Argentina (27)
C
0.70
0.86
1
0.96
0.97
0.99
0.04 substitutions per site
(c)
Z. querula
Z. albicollis
S. del Estero, Argentina (23)
Jujuy, Argentina (18)
Tucumán, Argentina (21)
Tucumán, Argentina (21)
Mendoza, Argentina (25)
Santa Cruz, Bolivia (16)
Santa Cruz, Bolivia (17)
Santa Cruz, Bolivia (17)
Santa Cruz, Bolivia (17)
Santa Cruz, Argentina (32)
T. del Fuego, Argentina (33)
T. del Fuego, Argentina (33)
T. del Fuego, Argentina (33)
T. del Fuego, Argentina (33)
Mendoza, Argentina (25)
Santa Cruz, Bolivia (16)
B
Buenos Aires, Argentina (27)
Buenos Aires, Argentina (27)
Buenos Aires, Argentina (27)
Buenos Aires, Argentina (27)
Corrientes, Argentina (24)
C
La Paz, Bolivia (15)
Pasco, Peru (13)
Huánuco, Peru (12)
Huánuco, Peru (12)
San José, Costa Rica (4)
San José, Costa Rica (4)
Aragua, Venezuela (6)
Aragua, Venezuela (6)
Aragua, Venezuela (6)
Aragua, Venezuela (6)
Aragua, Venezuela (6)
A
0.92
0.93
0.94
1
0.8
0.57
0.86
0.61
0.98
0.96
0.97
0.89
1
0.57
0.3 substitutions per site
(d)
Z. querula
Z. albicollis
San José, Costa Rica (4)
San José, Costa Rica (4)
Pasco, Peru (13)
Huánuco, Peru (12)
Huánuco, Peru (12)
Aragua, Venezuela (6)
Aragua, Venezuela (6)
Aragua, Venezuela (6)
Aragua, Venezuela (6)
Aragua, Venezuela (6)
A
La Paz, Bolivia (15)
S. del Estero, Argentina (23)
Jujuy, Argentina (18)
Tucumán, Argentina (21)
Tucumán, Argentina (21)
Santa Cruz, Argentina (32)
T. del Fuego, Argentina (33)
T. del Fuego, Argentina (33)
T. del Fuego, Argentina (33)
T. del Fuego, Argentina (33)
Buenos Aires, Argentina (27)
Buenos Aires, Argentina (27)
Buenos Aires, Argentina (27)
Buenos Aires, Argentina (27)
Mendoza, Argentina (25)
Mendoza, Argentina (25)
Corrientes, Argentina (24)
Santa Cruz, Bolivia (16)
Santa Cruz, Bolivia (16)
Santa Cruz, Bolivia (17)
Santa Cruz, Bolivia (17)
Santa Cruz, Bolivia (17)
B, C
1
0.55
0.98
0.2 substitutions per site
(e)
Z. querula
Z. albicollis
S. del Estero, Argentina (23)
Jujuy, Argentina (18)
Tucumán, Argentina (21)
Tucumán, Argentina (21)
Santa Cruz, Argentina (32)
T. del Fuego, Argentina (33)
T. del Fuego, Argentina (33)
T. del Fuego, Argentina (33)
T. del Fuego, Argentina (33)
Buenos Aires, Argentina (27)
Buenos Aires, Argentina (27)
Buenos Aires, Argentina (27)
Buenos Aires, Argentina (27)
Mendoza, Argentina (25)
Mendoza, Argentina (25)
Corrientes, Argentina (24)
La Paz, Bolivia (15)
Santa Cruz, Bolivia (16)
Santa Cruz, Bolivia (16)
San José, Costa Rica (4)
San José, Costa Rica (4)
Santa Cruz, Bolivia (17)
Santa Cruz, Bolivia (17)
Pasco, Peru (13)
Santa Cruz, Bolivia (17)
Huánuco, Peru (12)
Huánuco, Peru (12)
Aragua, Venezuela (6)
Aragua, Venezuela (6)
Aragua, Venezuela (6)
Aragua, Venezuela (6)
Aragua, Venezuela (6)
0.99
0.05 substitutions per site
(f)
Z. albicollis
S. del Estero, Argentina (23)
Jujuy, Argentina (18)
Tucumán, Argentina (21)
Tucumán, Argentina (21)
Santa Cruz, Argentina (32)
T. del Fuego, Argentina (33)
T. del Fuego, Argentina (33)
T. del Fuego, Argentina (33)
Buenos Aires, Argentina (27)
Buenos Aires, Argentina (27)
Buenos Aires, Argentina (27)
Buenos Aires, Argentina (27)
Mendoza, Argentina (25)
Corrientes, Argentina (24)
La Paz, Bolivia (15)
Santa Cruz, Bolivia (16)
Santa Cruz, Bolivia (16)
San José, Costa Rica (4)
San José, Costa Rica (4)
Santa Cruz, Bolivia (17)
Santa Cruz, Bolivia (17)
Pasco, Peru (13)
Huánuco, Peru (12)
Huánuco, Peru (12)
Aragua, Venezuela (6)
Aragua, Venezuela (6)
Aragua, Venezuela (6)
Aragua, Venezuela (6)
T. del Fuego, Argentina (33)
Mendoza, Argentina (25)
Santa Cruz, Bolivia (17)
Aragua, Venezuela (6)
0.99
0.88
0.1 substitutions per site
(g)
Z. querula
Z. albicollis
S. del Estero, Argentina (23)
Jujuy, Argentina (18)
Tucumán, Argentina (21)
Tucumán, Argentina (21)
Santa Cruz, Argentina (32)
T. del Fuego, Argentina (33)
Buenos Aires, Argentina (27)
Buenos Aires, Argentina (27)
Buenos Aires, Argentina (27)
Buenos Aires, Argentina (27)
Mendoza, Argentina (25)
Corrientes, Argentina (24)
Santa Cruz, Bolivia (16)
Santa Cruz, Bolivia (16)
San José, Costa Rica (4)
San José, Costa Rica (4)
Santa Cruz, Bolivia (17)
Santa Cruz, Bolivia (17)
Santa Cruz, Bolivia (17)
Huánuco, Peru (12)
Huánuco, Peru (12)
Aragua, Venezuela (6)
Aragua, Venezuela (6)
Aragua, Venezuela (6)
T. del Fuego, Argentina (33)
T. del Fuego, Argentina (33)
T. del Fuego, Argentina (33)
La Paz, Bolivia (15)
Pasco, Peru (13)
1
1
0.6
0.07 substitutions per site
