# Supplementary material for: Continental phylogeography of an ecologically and morphologically diverse Neotropical songbird, Zonotrichia capensis
Source: BMC Evol Biol. 2013 Mar 1;13:58. doi: 10.1186/1471-2148-13-58 (PMC3632491; doi:10.1186/1471-2148-13-58)
Supplement: Additional file 4 — Posterior density curves for splitting times, migration rates and effective population sizes estimated using IMa2. (PDF 236 kb) [file 1471-2148-13-58-S4.pdf]

Additional File 4

Posterior density curves for splitting times (a), bidirectional migration rates (b) and effective populations sizes (c) estimated using IMa2. (c) Note that the posterior probability curve for the effective population size of the ancestor of B and C does not reach zero in the upper bound of the prior. (d) Schematic representation of the relationship between lineages A, B and C (following Fig. 2) and the different parameters estimated in the isolation with migration model.

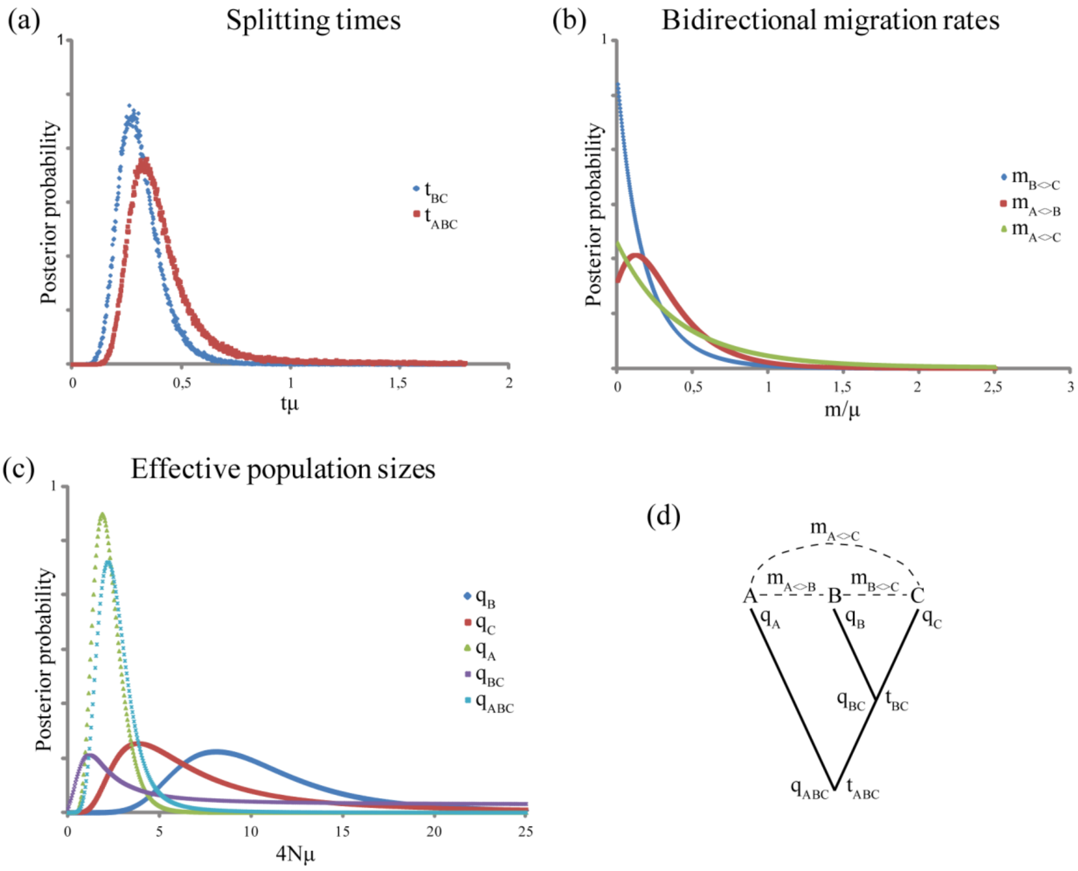
(a)
Splitting times
Posterior probability
t_{BC}
t_{ABC}
$t\mu$
0
0,5
1
1,5
2
(b)
Bidirectional migration rates
Posterior probability
$m_{B<>C}$
$m_{A<>B}$
$m_{A<>C}$
m/μ
0
0,5
1
1,5
2
2,5
3
(c)
Effective population sizes
Posterior probability
q_B
q_C
q_A
q_{BC}
q_{ABC}
$4N\mu$
0
5
10
15
20
25
(d)
$m_{A<>C}$
$m_{A<>B}$
$m_{B<>C}$
A
B
C
q_A
q_B
q_C
q_{BC}
t_{BC}
q_{ABC}
t_{ABC}
